# DNA damage repair mutations in pancreatic cancer– prognostic or predictive?

**DOI:** 10.3389/fonc.2023.1267577

**Published:** 2023-10-25

**Authors:** Ya-Fei Hu, Hai-Jie Hu, Heng-Chung Kung, Tian-Run Lv, Jun Yu, Fu-Yu Li

**Affiliations:** ^1^ Department of Biliary Surgery, West China Hospital of Sichuan University, Chengdu, Sichuan, China; ^2^ Krieger School of Arts and Sciences, Johns Hopkins University, Baltimore, MD, United States; ^3^ Department of Medicine, Johns Hopkins University School of Medicine, Baltimore, MD, United States; ^4^ Department of Oncology, Johns Hopkins University School of Medicine, Baltimore, MD, United States

**Keywords:** progression-free survival, overall survival, DNA damage repair gene, pancreatic cancer, platinum-based chemotherapy

## Abstract

**Objective:**

The efficacy of platinum-based chemotherapy (PtCh) for pancreatic cancer (PC) patients with DNA damage repair gene mutations (DDRm) compared to those without DDRm remains uncertain.

**Methods:**

After a thorough database searching in PubMed, Embase, and Web of Science, a total of 19 studies that met all the inclusion criteria were identified. The primary outcomes were overall survival (OS) and progression-free survival (PFS) for PC patients with DDRm versus those without DDRm after PtCh.

**Results:**

Patients with advanced-stage PC who have DDRm tend to have longer OS compared to patients without DDRm, regardless of their exposure to PtCh (HR=0.63; I^2^ = 66%). Further analyses indicated that the effectiveness of PtCh for OS was modified by DDRm (HR=0.48; I^2^ = 59%). After the first- line PtCh (1L-PtCh), the PFS of advanced-stage PC with DDRm was also significantly improved (HR=0.41; I^2^ = 0%). For patients with resected PC, regardless of their exposure to PtCh, the OS for patients with DDRm was comparable to those without DDRm (HR=0.82; I^2^ = 71%). Specifically, for patients with resected PC harboring DDRm who received PtCh (HR=0.85; I^2^ = 65%) and for those after non-PtCh (HR=0.87; I^2^ = 0%), the presence of DDRm did not show a significant association with longer OS.

**Conclusion:**

1L-PtCh treatment is correlated with favorable survival for advanced-stage PC patients with DDRm. For resected-stage PC harboring DDRm, adjuvant PtCh had limited effectiveness. The prognostic value of DDRm needs to be further verified by prospective randomized controlled trials.

**Systematic Review Registration:**

https://www.crd.york.ac.uk/prospero/, identifier CRD42022302275.

## 1 Introduction

Pancreatic cancer (PC) displaying a 5-year overall survival (OS) rate of <10% is a leading cause of cancer-related death worldwide (1). The standard therapeutic approach for patients with resectable PC involves curative surgical resection followed by adjuvant chemotherapy. Despite advancements in multiagent chemotherapy that have improved prognosis for advanced-stage of PC (2, 3), the mOS for patients with unresectable PC remains <1 year. Therefore, currently, available chemotherapy agents for advanced-stage PC demonstrate modest and/or limited effectiveness (4). As a result, there is an urgent need to gain a comprehensive understanding of the actionable molecular pathology features of PC for driving the development of novel and effective therapeutic approaches (2–5).

Pathogenic gene mutations have emerged as crucial contributors to PC development. In addition to well-established driver gene alterations such as KRAS and TP53 (6), recent investigations have elucidated that a notable proportion of PC patients (approximately 12%–25%) harbor mutations in genes involved in DNA damage repair (DDR) (7–9). These specific mutations can lead to disruptions in DNA homologous recombination (HR), resulting in deficiency and subsequent promotion of oncogenic processes in PC. In select Western cohorts (7), a notable disparity has been observed in the OS between PC patients with DDR gene mutations (DDRm) and those without DDRm. While other studies have yielded contrasting results, certain investigations have indicated that PC patients with DDRm may exhibit comparable or even worse prognoses than those without DDRm (10, 11). The prognostic value of DDRm in PC needs more confirmation. Principe et al. (12) conducted a study exploring the potential benefits of platinum-based chemotherapy (PtCh) in patients with PC who have DDRm. Their findings suggested that such patients may experience improved outcomes when treated with PtCh. Other literature additionally reported an augmented risk of disease progression or mortality in patients with DDRm who were exposed to non-first-line PtCh treatments (13, 14). These findings support that advanced-stage PC patients with DDRm may be sensitive to certain agents such as PtCh. However, the treatment efficacy of PtCh in resected-stage PC patients with DDRm remains uncertain according to existing literature (15, 16). Shun Yu et al. (17) suggested that PtCh may provide survival benefits for resected PC patients with pathogenic germline BRCA/PALB2 mutations, whereas Blair et al. (18) reported worse survival outcomes in BRCA-mutated carriers compared to wild-type counterparts after PtCh treatment. These divergent results indicate that the implications of DDRm for different stages of PC may vary and need further confirmation (7, 19–21).

DDRm genes are not tested routinely worldwide; thus, previously published studies investigating DDRm in PC have predominantly utilized small sample sizes and included patients at various stages of the disease. Consequently, a notable degree of heterogeneity exists within these studies (12, 13, 15). To address this inherent heterogeneity and consolidate the available evidence, we conducted a systematic review of the published literature focused on comparing the prognosis of advanced and resected-stage PC patients with DDRm versus those without DDRm.

## 2 Materials and methods

We performed the meta-analyses according to the Preferred Reporting Items for Systematic Reviews and Meta-Analyses (PRISMA) statement (22). The study protocol was published on PROSPERO (number CRD42020146320). Two authors (YF Hu and HJ Hu) independently screened the titles and abstracts of studies to identify literature that met all inclusion criteria. A third reviewer (Fu-Yu Li) was consulted when any disagreements were met. The Newcastle–Ottawa Scale (23) was used for quality evaluation of cohort or case–control studies; studies were selected if they had a score above 5.

### 2.1 Search details

We searched relevant studies published in PubMed, Embase, and Web of Science after 2015. The search strategy used a combination of Medical Subject Heading terms (MESH terms) including pancreatic cancer, DNA damage repair, Platinum, and their related words. The following search strategy was used “((((((((((Pancreatic cancer[MeSH Terms]) OR (pancreatic Neoplasms[Text Word])) OR (pancreatic neoplasm*[Text Word])) OR (pancreatic neoplasm*[Text Word])) OR (pancreatic carcinoma[Text Word])) OR (pancreatic adenocarcinoma[Text Word])) AND (DNA damage repair[MeSH Terms])) OR (DNA damage response[Text Word])) AND (Platinum[MeSH Terms])) OR (PtCh[Text Word])”. Word variations have also been searched. The search strategy was adapted according to each database configuration.

### 2.2 Inclusion criteria

We only included studies of high quality and met all our inclusion criteria as follows:

1) Patients: confirmed PC malignancy of resected or advanced stage.
2) Interventions: PtCh vs. non-PtCh.
3) Comparators: PC with germline or somatic DDRm versus those without DDRm or wild type.
4) Primary outcomes: including OS or PFS.
5) Study type: comparative studies on humans and of English languages.

### 2.3 Exclusion criteria

Exclusion criteria included the following:

a) Patients with benign pancreatic diseases or unconfirmed PC.
b) Did not provide any survival outcomes.
c) Included fewer than three patients or a case report.
d) Designed as single-arm and/or only included patients with DDRm genes.

### 2.4 Data extraction and quality assessments

Two reviewers (Hu YF and Hu HJ) independently extracted the following information from the selected studies: author, publication year, study type, patient characteristics, PC stages, interventions, mutation types (germline/somatic), details, and primary conclusions (**Table 1**). The study quality was scaled by the NOS score measurement, and PFS/OS in DDRm vs. without DDRm groups after the first or second/later line of PtCh was also presented (**Supplementary Material**).

**Table 1:** Studies included in the systematic review.

Author/year	Study type	Main outcomes	Num.of DDRm versus without DDRm	Interventions	Gene mutation type of the included patients	Details	Main conclusions	NOS score
Max/2020	Retrospective	PFS/ORR	Advanced-stage PC 26 versus 52	All PtCh	Germline	Patients with non-DDRm the only observed responses were to FOLFIRINOX	PDAC with DDRm had a high ORR and prolonged PFS to PtCh chemotherapy.	7
Kim/2018	Retrospective	OS	Advanced-stage PC 29 versus 58	PtCh/Non-PtCh	Germline	In patients not treated with PtCh, there was no difference in OS between DDRm versus wild groups.	PtCh resulted survival benefits for advanced PDAC with germline BRCA1/BRCA2/PALB2 mutations.	7
Park/2020	Retrospective	PFS/OS	Advanced-stage PC 50 versus 212	All PtCh	Germline/somatic	Advanced-stage PDAC patients with HRD had improved OS regardless of their 1L-treatments but most with PtCh	Pathogenic HRD in PDAC with the best outcome when treated with 1L- PtCh.	8
Kondo/2018	Retrospective	mPFS	Advanced-stage PC 8 versus 9	FOLFIRINOX/Oxaliplatin-based chemotherapy	Germline/somatic	Two patients with inactivating HRR-related gene mutations receiving 1L-FOLFIRINOX had PFS>24months.	Inactivating HRR-related gene mutations are predictive of response to Oxaliplatin-based chemotherapy in patients with PDAC	7
Sofia/2019	Retrospective	mOS/PFS	Metastatic PC 9 versus 40	1L-FOLFIRINOX	Germline/somatic	No deaths in germline pathogenic DDRm patients treated with frontline FOLFIRINOX.	DDRm as a predictive biomarker for FOLFIRINOX benefits and superior PFS were seen after treated with the platinum containing regimen FOLFIRINOX	7
Sehdev/2018	Retrospective	OS	Metastatic PC 12 versus 24	1L-FOLFIRINOX	Germline/somatic	51.4% had any family history of cancers. presence of DDRm was associated with improved OS.	DDRm are associated with improved OS in PDAC patients treated with FOLFIRINOX.	8
Yurgelun/2019	Retrospective	OS/DFS	Resected PC 28 versus 261	Resection/PtCh	Germline/somatic	Patients with germline DDRm had superior survival.	Compared to non-carriers, individuals with germline DDRm had superior survival after PDAC resection.	8
Chang/2022	Retrospective	OS	Resected PC 23 versus 57	Resection/PtCh	Germline	Patients carrying any HRD, most platinum-naïve, had comparable survival with those with wild-type tumors	No prognostic effect from BRCA1/2/PALB2 or other HR-DNA damage repair gene defects for resected PDAC patients.	7
Alex/2018	Retrospective Case-control	OS/DFS	Resected PC 22 versus 105	Resection/PtCh/Non-PtCh	Germline	Resected PDAC with BRCA1/2 mutations had worse survival after surgery.	PtCh were associated with markedly improved survival in patients with BRCA1/2 mutations, with survival differences comparable to wild-type patients.	7
Shun Yu/2019	Retrospective	mOS	Resected PC 32 versus 64	Resection/PtCh	Germline	PDAC with DDRm received perioperative PtCh had improved mOS compared with those who did not	PtCh may confer survival benefits in resected and pathogenic germline BRCA/PALB2 mutation PDAC	7
Golan/2017	Retrospective	DFS/OS	Resected PC 25 versus 49	Resection/PtCh	Germline	81.7% (n=58) patients had any family history of malignancies	Superior OS was observed for BRCA-associated advanced PDAC with PtCh	7
Hu/2020	Retrospective	DFS/OS	Resected PC 19 versus 375	Resection/PtCh	Germline/somatic	Patients were divided into 3 groups according to the mutation types.	DDRm confer survival benefits to sporadic PDAC patients.	7
Marina/2022	Retrospective	OS/PFS	All stages PC	1L-PtCh vs. non- PtCh	Germline/somatic	No prognostic value was observed for resected PC patients with BRCA1/2, PALB2, or other HR/FA genes mutations.	BRCA1/2 and PALB2 genes mutations increase the sensitivity of PtCh to PC.	7
Hannan/2021	Retrospective	mOS	All stages PC	PtCh/Non-PtCh	Germline/somatic	Including PDAC with known somatic/germline ATM alteration	PC patients with pathogenic ATM alterations had improved outcomes.	7
Elena/2021	Retrospective	OS	All stages PC	PtCh	Germline/somatic	Cancer-associated and HRR genes were both identified in European PDAC patients	The presence of P/LPVs in HRR genes did not predict benefit from PtCh	8
Lin Shui/2020	Retrospective	OS	All stages PC	PtCh/Olaparib/PD-1	Germline/somatic	Baseline characteristics of overall patients were comparable.	Germline and somatic DDR mutation may predict the Olaparib/PtCh efficacy in Chinese populations with PDAC	7
Pishvaian/2019	Retrospective	mOS	All stages PC	PtCh/Non-PtCh	Germline/somatic	DDRm patients were divided into 3 group based on the pathogenic mutation types	HR-DDRmt patients receive the benefit of PtCh treatment; mOS was similar in all resected PDAC.	7
Yadav/2020	Prospective	OS	All stages PC	Surgery/chemotherapy/No Chemotherapy/missing	Germline/somatic	Germline ATM mutation carriers had longer OS vs. non-carriers	Germline DDRm PDAC had longer OS compared with non-carriers.	8
Pishvaian/2020	Retrospective	mOS	All stages PC	PtCh/Non-PtCh/matched therapy	Germline/somatic	Patients received matched therapy according to actionable molecular mutations	Patients received two or more lines of therapy; the matched therapy group had a significantly longer median OS than the unmatched therapy gru	7

HRD, homologous recombination deficiency; DDRm, DNA-damage-repair mutated genes; P/LPVs, pathogenic/likely pathogenic variants; PtCh, platinum-based chemotherapy; PFS, progression-free survival; ORR, objective response rate; RR, response rate; DCR, disease control rate; OS, overall survival; ITT, intention-to-treat analysis;1L-platinum, first-line platinum-based therapy; PC, pancreatic cancer; PDAC, pancreatic ductal adenocarcinoma; PGAs, pathogenic germline alterations. CisP-Gem, Gemcitabine and Cisplatin; all stages, resectable or advanced stages of PC; PC, pancreatic cancer.

### 2.5 Statistical methods

Our meta-analysis was performed with Review Manager (computer program) V.5.4 (Nordic Cochrane Center, Copenhagen) and Stata 14.0. Hazard ratio (HR) was presented as a risk ratio of compared groups with 95% certification interval (CI). The I*^2^* test was used to confirm the homogeneity among the study results. When research results with low statistically significant heterogeneity (I^2^ ≤ 60%) were found, a fixed-effect model was used. Funnel-shaped graphs would be constructed if there were more than 10 studies in the analyses. Sensitivity analyses were performed on the results with multiple methods, including study exclusion.

## 3 Results

**Figure 1:**
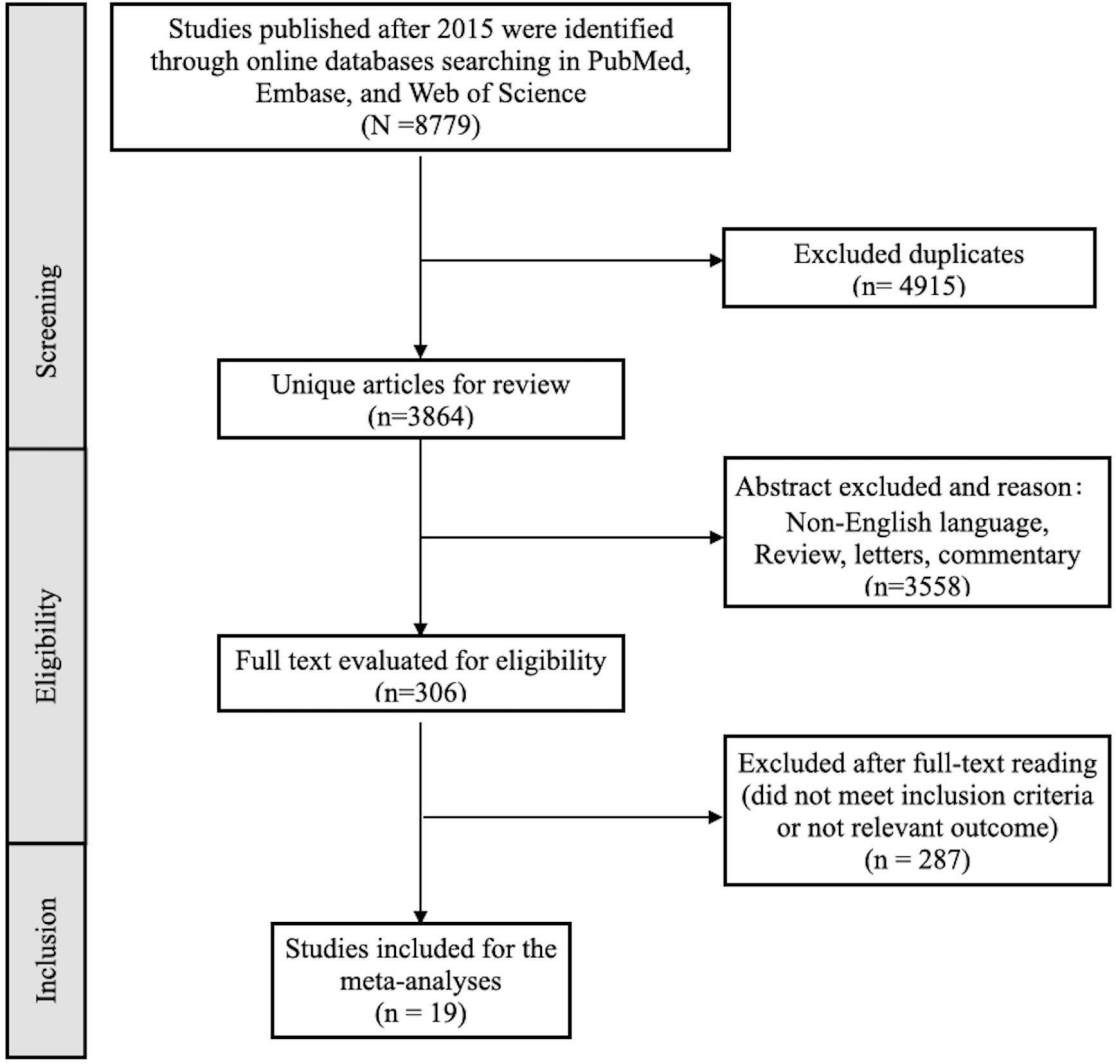
The flowchart of the study selection process.

The flowchart of the study selection process is reported in **Figure 1**. After conducting a thorough databases searching including PubMed, Embase, and Web of Science, we identified a total of 8,779 relevant studies published after 2015. We eliminated 4,915 duplicate studies, leaving us with 3,864 studies for review. After screening abstract and titles, 3,558 studies were judged not relevant with reasons of non-English language literature, reviews, letters, or animal studies. After screening full texts of 306 studies, 287 studies that did not meet all the inclusion criteria were excluded. Consequently, a total of 19 studies that compared the effectiveness of PtCh for PC patients with DDRm versus those without DDRm were included in the meta-analyses (10, 11, 14, 16–20, 24–34).

The primary outcomes of the study focused on OS and PFS in patients with advanced/resected PC who had DDRm compared to those without DDRm. The secondary outcomes of the study were PFS for advanced-stage PC following various lines of PtCh and mOS improvement for advanced-stage PC who had mutated ATM/ATR genes compared to a control group with wild-type genes. The findings of our meta-analyses are presented in **Table 2**.

**Table 2:** Primary and Secondary outcomes of the meta-analyses.

Subgroups	Outcome Index	Number. Of Studies	Statistical Method	Effect Estimate	p-Value	Heterogeneity
Primary outcomes						
Resected PC						
PtCh for DDRm vs. without DDRm	OS	5	HR (IV, Random, 95% CI)	0.85(0.64, 1.13)	p=0.27	I^2^ = 65%
Non-PtCh for DDRm vs. without DDRm	OS	3	HR (IV, Fixed, 95% CI)	0.87(0.37, 2.00)	p=0.74	I^2^ = 0%
DDRm vs. without DDRm	OS	9	HR (IV, Random, 95% CI)	0.82 (0.65, 1.03)	p=0.09	I^2^ = 71%
Advanced PC						
PtCh for DDRm vs. without DDRm	PFS	4	HR (IV, Fixed, 95% CI)	0.41(0.30, 0.56)	p<0.00001	I^2^ = 0%
PtCh for DDRm vs. without DDRm	OS	5	HR (IV, Random, 95% CI)	0.48(0.32, 0.71)	p=0.0002	I^2^ = 59%
Non-PtCh for DDRm vs. without DDRm	OS	2	HR (IV, Random, 95% CI)	0.95(0.33, 2.71)	p=0.92	I^2^ = 78%
DDRm vs. without DDRm	OS	6	HR (IV, Random, 95% CI)	0.63 (0.47,0.87)	p=0.004	I^2^ = 66%
Secondary outcomes for advanced PC						
First-line PtCh for DDRm vs. without DDRm	PFS	3	HR (IV, Random, 95% CI)	0.44 (0.32, 0.59)	p<0.00001	I^2^ = 0%
Second or later line PtCh for DDRm vs. without DDRm	PFS	3	HR (IV, Random, 95% CI)	0.98 (0.51, 1.87)	p=0.95	I^2^ = 83%
ATM/ATR mutations vs. wild control group	OS	2	HR (IV, Random, 95% CI)	0.46 (0.14, 1.52)	p=0.20	I^2^ = 68%

HR, hazard ratio; 95%CI, 95% certification interval; DDRm, DNA-damage-repair mutations; Fixed/Random, fixed/random-effects model; PFS, progression-free survival; PC, pancreatic cancer; PtCh, platinum-based therapy; First-line, first-line PtCh; OS, overall survival; mOS, median OS.

### 3.1 Primary outcomes

#### 3.1.1 Prognostic value of DDR

**Figure 2:**
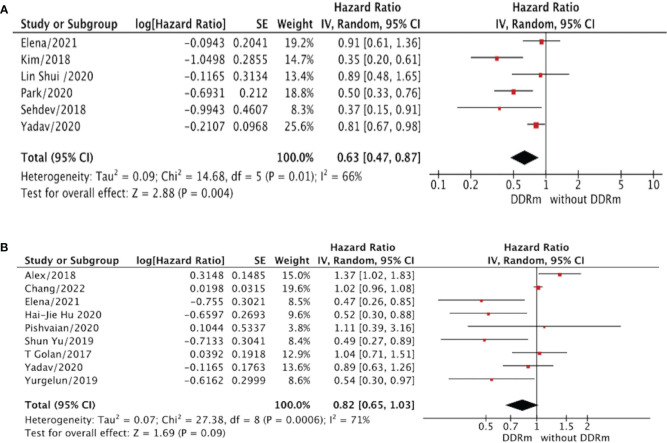
**(A)** The overall survival (OS) for advanced PC patients (DDRm vs. without DDRm). **(B)** The overall survival (OS) for resected PC patients (DDRm vs. without DDRm).

To evaluate the predictive role of DDRm on OS in patients with PC, we conducted an analysis of OS between patients with DDRm and those without DDRm, irrespective of the treatment approach employed (**Figures 2A, B**). For patients with advanced PC and DDRm, we observed a significantly longer OS in comparison to those without DDRm, with a hazard ratio (HR) of 0.63 (95% confidence interval [CI], 0.47–0.87; p=0.004) (**Figure 2A**). However, for resected stages of PC, the presence of DDRm genes did not demonstrate any significant OS benefits, with an HR of 0.82 (95% CI, 0.65–1.03; p=0.09) (**Figure 2B**). It is important to note that both the analyses conducted in advanced and resected PC patients exhibited notable heterogeneity, with I^2^ values of 66% and 71%, respectively, indicating substantial variability among the included studies.

#### 3.1.2 Therapeutic value of DDRm

**Figure 3:**
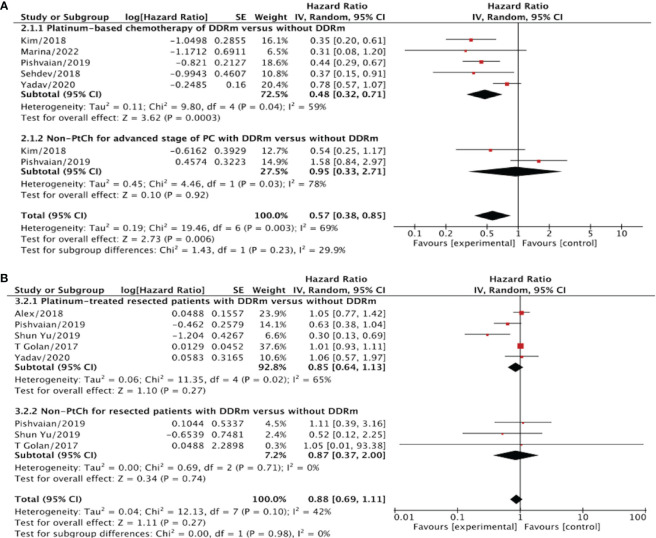
**(A)** The overall survival (OS) for advanced/metastatic PC patients without DDRm versus without DDRm after PtCh or non- PtCh. **(B)** The overall survival for early/resected PC patients with DDRm versus without DDRm after PtCh or non- PtCh.

**Figure 4:**
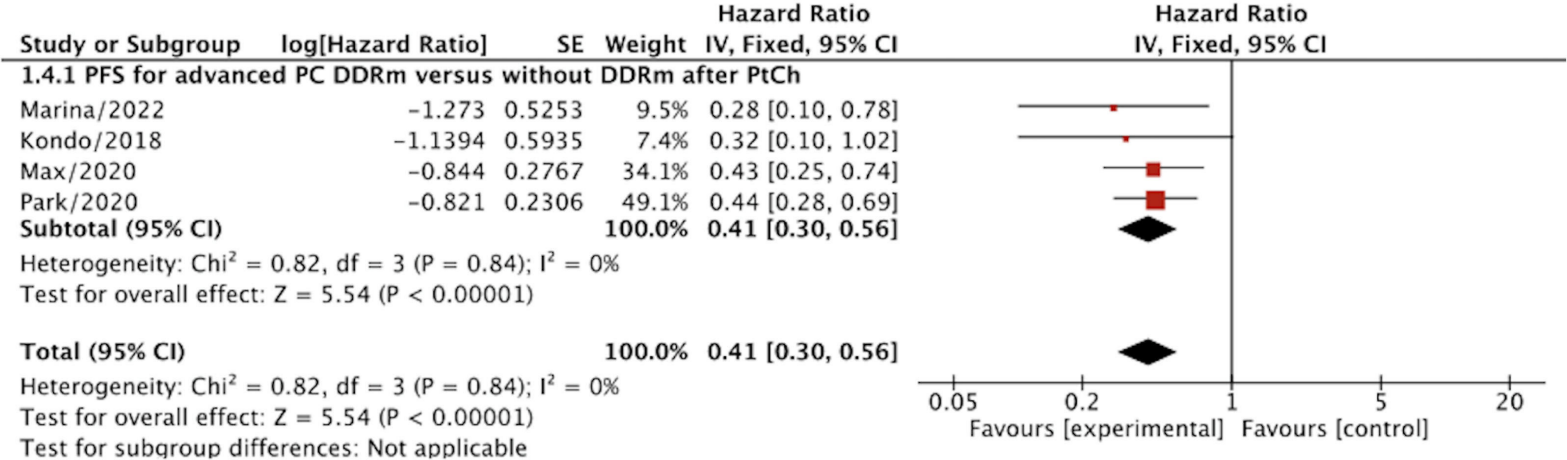
The progression-free survival (PFS) for advanced PC patients with DDRm versus without DDRm after PtCh.

To address the observed significant heterogeneity in our results, we conducted a subgroup analysis to evaluate the impact of DDRm in PC patients based on the type of chemotherapy received, specifically PtCh versus non-PtCh treatment. For this analysis, we included patients who received PtCh at any stage following the diagnosis of PC. We focused on the OS/PFS differences among the two treatment groups. The findings of this subgroup analysis are presented in **Figures 3** and **4** of our manuscript.

#### 3.1.3 OS for advanced/resected-stage PC patients after PtCh

Our analysis revealed that the presence of DDRm is associated with improved OS in advanced PC patients who received PtCh, with an HR of 0.48 (95% CI, 0.32–0.71; p=0.0003) compared to patients without DDRm (**Figure 3A**). However, in the case of resected PC patients with DDRm genes who received PtCh, the presence of DDRm did not show a significant association with longer OS, as the HR was 0.85 (95% CI, 0.64–1.13; p=0.27) (**Figure 3B**). It is worth noting that no significant heterogeneity was detected in these analyses, with I^2^ values of 59% and 65% for advanced and resected PC patients, respectively.

#### 3.1.4 OS for advanced/resected-stage PC patients after non-PtCh

We evaluated survival outcomes in PC Patients with non-PtCh treatment to evaluate the prognostic value of DDRm. These patients were divided into resected and advanced stages (**Figures 3A, B**). Our analysis did not reveal any statistically significant differences in OS between patients with DDRm and those without DDRm in either the resected or advanced subgroups after non-PtCh treatment. Specifically, among patients with advanced PC and DDRm after non-PtCh treatment, the OS was comparable to patients without DDRm, with an HR of 0.95 (95% CI, 0.33–2.71; I^2^ = 78%; p=0.92) (**Figure 3A**). Similarly, for patients with resected PC and DDRm after non-PtCh, the pooled HR was 0.87 (95% CI, 0.37–2.00; p=0.74) (**Figure 3B**). Importantly, there was no heterogeneity detected in the comparison (I^2^ = 0%). Based on these results, it appears that the DDR status does not carry a definitive prognostic value for patients who did not undergo PtCh treatment.

#### 3.1.5 PFS for advanced PC patients after PtCh

The value of DDRm for advanced PC after PtCh could also be demonstrated by an increased PFS with HR=0.41 (95% CI, 0.30–0.56; p<0.00001) compared to the without DDRm. The analyses were presented with no heterogeneity (I^2^ = 0%) (**Figure 4**).

### 3.2 Secondary outcomes

#### 3.2.1 PFS for advanced PC patients after the first/later line of PtCh

For patients with advanced PC, the association between DDRm and PtCh had been demonstrated by an increased PFS, which was only significantly observed in the first-line PtCh setting with HR=0.44 (95% CI, 0.32–0.59; p<0.00001; I^2^ = 0%). No significant difference was observed for PC in DDRm versus without DDRm group after the second/later line of PtCh (HR=0.98; 95% CI, 0.51–1.87; p=0.95; I^2^ = 83%) (**Supplementary Figure S1**).

#### 3.2.2 OS for advanced PC patients with ATM versus wild control groups

The further gene-level analysis demonstrated that germline/somatic ATM/ATR mutation carriers had comparable OS to patients without those mutations (HR=0.46; 95%CI, 0.14–1.52; p=0.20), with moderate heterogeneity (I^2^ = 68%) (**Supplementary Figure S2**).

## 4 Sensitivity analyses

### 4.1 OS for advanced-stage PC with DDRm vs. without DDRm regardless of adjuvant therapy methods

We excluded one study by Kim et al. for some patients in the cohort who did not receive any therapy. Patients with advanced tumors and harboring DDRm were found to be positively associated with a significantly longer OS after chemotherapy (HR=0.72; 95% CI, 0.55–0.94; p=0.01) with low heterogeneity in the results (I^2^ = 49%) (**Supplementary Figure S3**).

### 4.2 OS for resected PC with DDRm vs. without DDRm regardless of adjuvant therapy methods

We excluded the studies by Chang et al. and Alex et al., as the studies included patients who did not receive any adjuvant therapy. After analysis, we could find longer OS for resected PC patients with DDRm (HR=0.73; 95%CI, 0.61–0.88; p=0.001) with low heterogeneity in the results (I^2^ = 49%) (**Supplementary Figure S4**).

## 5 Discussion

The clinical significance of DDRm cannot be overstated. Extensive research has shown that platinum-containing chemotherapy regimens exhibit enhanced efficacy against breast and ovarian cancer cells harboring DDRm (35–39). In PC, approximately 20% of patients carry DDRm. However, the relevance of DDRm in PC remains a topic of debate. Conflicting findings have emerged from studies investigating the association between DDRm and survival outcomes in PC patients, with some reports suggesting improved survival in DDRm individuals while others indicate comparable or even worse prognoses. Consequently, further investigation is imperative to ascertain the impact of DDRm on PC patients’ survival. Moreover, the underlying reasons for the observed survival benefits, whether attributed to the inherent prognostic advantage of DDRm or the therapeutic value in terms of response to PtCh, remain unclear. To address these questions, we undertook an investigation into the association between DDRm and survival prognosis in PC.

In this study, patients were classified into two categories based on PC stage: resected versus advanced. Additionally, patients were categorized according to the type of chemotherapy received: PtCh versus non-PtCh. Furthermore, patients were evaluated based on their DDRm: DDRm versus without DDRm. Our study demonstrated a significant improvement in OS specifically among the subset of advanced DDRm PC patients following PtCh. However, it was observed that only first-line PtCh resulted in superior PFS outcomes for these patients. Consistent with our findings, previous studies (13, 14) have also reported survival benefits for advanced DDRm PC patients compared to patients without DDRm when treated with PtCh. Interestingly, there was even an indication of a potential trend towards poorer prognostic outcomes for advanced DDRm PC patients receiving non-PtCh, in comparison to PC patients without DDRm (32).

Patients with advanced PC and DDRm demonstrated longer survival when exposed to PtCh compared to advanced PC patients without DDRm. These findings suggest that DDRm may hold predictive value in determining the efficacy of PtCh therapy for advanced PC.

In our analysis, we also investigated the survival difference between patients DDRm and those without DDRm who were treated with non-PtCh. Interestingly, we found that the OS outcomes were comparable between DDRm and without DDRm patients, both in the advanced and early-stage PC. These results indicate that while DDRm may possess some predictive value in selecting patients for PtCh therapy in specific cases of PC, the pure prognostic value of DDRm appears to be limited and necessitates further validation and confirmation through additional research.

In our study, we observed that advanced PC patients DDRm who received first-line platinum-based chemotherapy (1L-PtCh) experienced significantly prolonged PFS. However, no improvements in PFS were observed in the second or later treatment settings of the platinum-based chemotherapy approach. This suggests that 1L-PtCh may be the optimal treatment choice for advanced DDRm PC patients.

A study by Park et al. (14). also supports our findings, demonstrating that patients with pathogenic homologous recombination deficiency (HRD) in pancreatic cancer exhibited improved outcomes only when treated with 1L-PtCh. These findings further emphasize the potential benefits of 1L-PtCh as a treatment strategy for advanced DDRm PC patients.

The implications of our findings also extend to future clinical trial design, highlighting the significance of early germline testing in patients diagnosed with advanced PC. Early identification of DDRm (DDRm) through germline testing can aid in identifying patients who may derive benefits from 1L-PtCh and optimize treatment strategies tailored to their specific genetic profile. These insights contribute to the ongoing efforts to enhance precision medicine approaches in the treatment of pancreatic cancer.

In our study, we also investigated the survival outcomes of resected DDRm PC patients after adjuvant chemotherapy. We observed limited survival advantages for selected patients after adjuvant chemotherapy. When comparing resected DDRm PC patients to those without DDRm, neither PtCh nor non PtCh was associated with longer OS. However, it is worth mentioning that Shun Yu et al. (17) reported that perioperative PtCh in PC patients with DDRm resulted in improved mOS compared to those who did not receive perioperative PtCh. This suggests that certain adjuvant chemotherapy regimens may confer benefits to resected DDRm PC patients. Nevertheless, our analysis did not demonstrate a significant effect of DDRm on OS in resected PC patients, as comparable survival outcomes were observed between DDRm and without DDRm groups treated with either PtCh or non-PtCh.

It is important to note that Golan et al. (24) also reported no survival differences between BRCA mutation carriers and sporadic tumors. These findings highlight the necessity for more prospective studies to confirm these observations and further investigate the potential impact of DDRm on the outcomes of resected PC patients.

In conclusion, additional research is warranted to better understand the role of DDRm in resected PC patients and to elucidate the significance of surgical-related factors such as surgical margin and nodal status in determining outcomes. Prospective studies will provide more comprehensive insights into the impact of DDRm on survival outcomes and help refine treatment strategies for patients with resected PC.

Gemcitabine/nab-paclitaxel and FOLFIRINOX are the two primary first-line regimens utilized for the treatment of advanced-stage PC. Previous studies have reported an mOS of approximately 8.5 months for PC patients treated with gemcitabine/nab-paclitaxel, compared to 14 months for those treated with FOLFIRINOX (11, 26, 29, 40, 41). More recently, a study involving a smaller cohort of PC patients with DDRm genes treated with FOLFIRINOX demonstrated an improved OS (11). Currently, there are no established predictive biomarkers to identify patients who would benefit more from FOLFIRINOX treatment. However, further studies comparing the effectiveness of gemcitabine/nab-paclitaxel versus FOLFIRINOX in PC patients with DDRm genes could potentially establish DDRm as a valuable predictive biomarker for guiding decisions regarding FOLFIRINOX treatment.

Molecular studies involving sporadic PC have identified a complex mutational profile, and multiple genes have been reported to be associated with HR deficiency, but the exact related genes are not clear (5, 39, 42, 43). Additionally, not all DDRm genes play a role in the prognosis of PC. In our study, we found limited prognostic impact of the HRD-related gene *ATM* in PC patients, and few studies have evaluated the role of other DDRm genes. As such, we lack sufficient data to compare the prognostic impact of different types of DDRm on PC patients and the sensitivity of PC patients carrying different DDRm to PtCh therapy (2, 13, 21).

Of note, our study had several important limitations. First, most of the included studies were retrospective, and selective bias was exciting. Furthermore, the samples of some studies were small, limiting the reliability of the conclusions. Second, the basic characteristics of the included patients do not completely match, which is inevitable in meta-analyses. Third, several rarer candidate DDR genes (e.g., *ATR*, *ATRX*, *CHEK1*, *RAD51L1*, and *RAD51L3*) were excluded from some of our included studies; therefore, some patients in the wild group may also have undetected DDRm genes, which may influence the outcomes. Finally, the targeted-sequencing approaches and the mutation status (somatic or germline) were also different. In conclusion, large-scale prospective randomized controlled studies are needed to confirm the benefits of PtCh treatment for PC patients with DDRm.

## 6 Conclusions

In our study, we observed an improved survival among patients with advanced PC who had DDRm after receiving PtCh. However, the effectiveness of PtCh on survival for resected DDRm PC patients was limited. Overall, our analysis did not demonstrate a significant prognostic effect of DDRm in PC patients. Nevertheless, our findings suggest that optimal therapy for advanced PC patients with DDRm may involve the use of a platinum-containing regimen. It is important to note that while appropriate chemotherapy for resected DDRm PC patients may result in a longer OS, surgery remains the only curative approach. These findings underscore the potential value of early germline testing in individuals diagnosed with PC, as it may provide insights into DDRm and guide treatment decisions. Moreover, given the limited data available, future studies should focus on assessing the variations in tumor biology and response to standard treatments among PC patients with different DDRm profiles.

## Data availability statement

The original contributions presented in the study are included in the article/**Supplementary Material**. Further inquiries can be directed to the corresponding author.

## Author contributions

Y-FH: Conceptualization, Data curation, Formal Analysis, Investigation, Methodology, Project administration, Software, Supervision, Validation, Writing – original draft, Writing – review & editing. H-JH: Conceptualization, Data curation, Formal Analysis, Investigation, Methodology, Project administration, Software, Supervision, Validation, Writing – original draft, Writing – review & editing. H-CK: Conceptualization, Data curation, Investigation, Methodology, Software, Writing – original draft. T-RL: Conceptualization, Data curation, Investigation, Software, Writing – original draft. JY: Conceptualization, Data curation, Funding acquisition, Investigation, Methodology, Resources, Software, Visualization, Writing – original draft. F-YL: Conceptualization, Funding acquisition, Investigation, Resources, Software, Visualization, Writing – original draft.
