# Supplementary material for: DNA damage repair mutations in pancreatic cancer– prognostic or predictive?
Source: Front Oncol. 2023 Oct 25;13:1267577. doi: 10.3389/fonc.2023.1267577 (PMC10634423; doi:10.3389/fonc.2023.1267577)

Supplementary Fig 1. The PFS of advanced PC patients after different lines of PtCh therapy.

Study or Subgroup	log[Hazard Ratio]	SE	Weight	Hazard Ratio IV, Random, 95% CI
5.1.1 First-line				
Marina/2022	-1.273	0.5253	12.4%	0.28 [0.10, 0.78]
Park/2020	-0.821	0.2306	17.8%	0.44 [0.28, 0.69]
Pishvaian/2019	-0.755	0.2291	17.8%	0.47 [0.30, 0.74]
Subtotal (95% CI)			47.9%	0.44 [0.32, 0.59]
Heterogeneity: Tau² = 0.00; Chi² = 0.82, df = 2 (P = 0.66); I² = 0%				
Test for overall effect: Z = 5.35 (P < 0.00001)				
5.1.2 Second or later				
Max/2020	0.3075	0.079	19.5%	1.36 [1.16, 1.59]
Park/2020	0.3507	0.2928	16.7%	1.42 [0.80, 2.52]
Pishvaian/2019	-0.8916	0.3414	15.8%	0.41 [0.21, 0.80]
Subtotal (95% CI)			52.1%	0.98 [0.51, 1.87]
Heterogeneity: Tau² = 0.27; Chi² = 11.83, df = 2 (P = 0.003); I² = 83%				
Test for overall effect: Z = 0.07 (P = 0.95)				
Total (95% CI)			100.0%	0.63 [0.35, 1.14]
Heterogeneity: Tau² = 0.46; Chi² = 52.24, df = 5 (P < 0.00001); I² = 90%				
Test for overall effect: Z = 1.52 (P = 0.13)				
Test for subgroup differences: Chi² = 4.89, df = 1 (P = 0.03), I² = 79.6%				

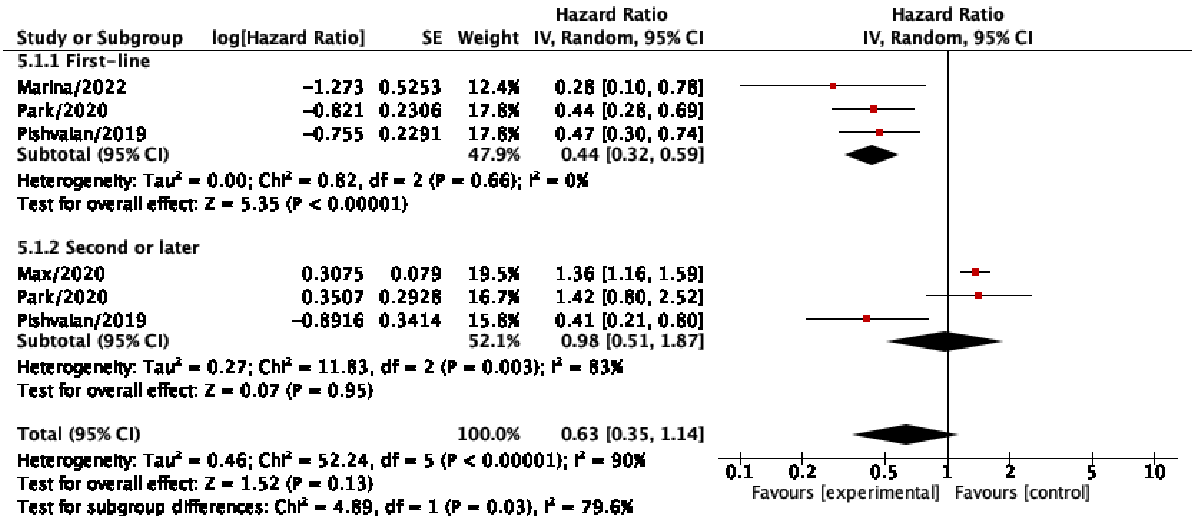

Supplementary Fig 2. The median OS for advanced PC with ATM/ATR vs wild control groups.

Study or Subgroup	log[Hazard Ratio]	SE	Weight	Hazard Ratio IV, Random, 95% CI
Hannan/2021	-1.6094	0.7073	35.3%	0.20 [0.05, 0.80]
Yadav/2020	-0.3285	0.1374	64.7%	0.72 [0.55, 0.94]
Total (95% CI)			100.0%	0.46 [0.14, 1.52]
Heterogeneity: Tau² = 0.56; Chi² = 3.16, df = 1 (P = 0.08); I² = 68%				
Test for overall effect: Z = 1.28 (P = 0.20)				

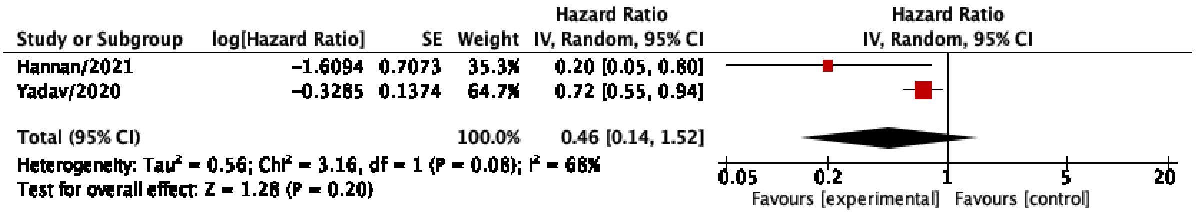

Supplementary Fig 3. Sensitivity analyses of the OS for advanced stage of PC with DDRm vs wild control groups after therapy.

Study or Subgroup	log[Hazard Ratio]	SE	Weight	Hazard Ratio IV, Random, 95% CI
Elena/2021	−0.0943	0.2041	22.1%	0.91 [0.61, 1.36]
Kim/2018	−1.0498	0.2855	0.0%	0.35 [0.20, 0.61]
Lin Shui /2020	−0.1165	0.3134	13.1%	0.89 [0.48, 1.65]
Park/2020	−0.6931	0.212	21.3%	0.50 [0.33, 0.76]
Sehdev/2018	−0.9943	0.4607	7.2%	0.37 [0.15, 0.91]
Yadav/2020	−0.2107	0.0968	36.3%	0.81 [0.67, 0.98]
Total (95% CI)			100.0%	0.72 [0.55, 0.94]

Heterogeneity: $Tau^2 = 0.04$; $Chi^2 = 7.81$, df = 4 (P = 0.10); $I^2 = 49\%$

Test for overall effect: Z = 2.45 (P = 0.01)

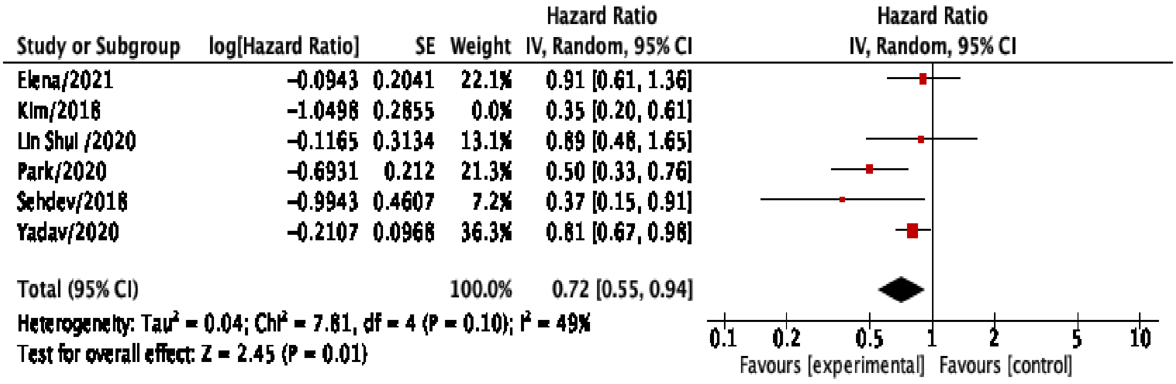

Supplementary Fig 4. Sensitivity analyses of the OS for resected stage of PC with DDRm vs wild control groups after therapy.

Study or Subgroup	log[Hazard Ratio]	SE	Weight	Hazard Ratio IV, Fixed, 95% CI
Alex/2018	0.3148	0.1485	0.0%	1.37 [1.02, 1.83]
Chang/2022	0.0198	0.0315	0.0%	1.02 [0.96, 1.08]
Elena/2021	−0.755	0.3021	10.0%	0.47 [0.26, 0.85]
Hai-Jie Hu 2020	−0.6597	0.2693	12.6%	0.52 [0.30, 0.88]
Pishvaian/2020	0.1044	0.5337	3.2%	1.11 [0.39, 3.16]
Shun Yu/2019	−0.7133	0.3041	9.9%	0.49 [0.27, 0.89]
T Golan/2017	0.0392	0.1918	24.8%	1.04 [0.71, 1.51]
Yadav/2020	−0.1165	0.1763	29.4%	0.89 [0.63, 1.26]
Yurgelun/2019	−0.6162	0.2999	10.1%	0.54 [0.30, 0.97]
Total (95% CI)			100.0%	0.73 [0.61, 0.88]

Heterogeneity: $Chi^2 = 11.78$, df = 6 (P = 0.07); $I^2 = 49\%$

Test for overall effect: Z = 3.27 (P = 0.001)

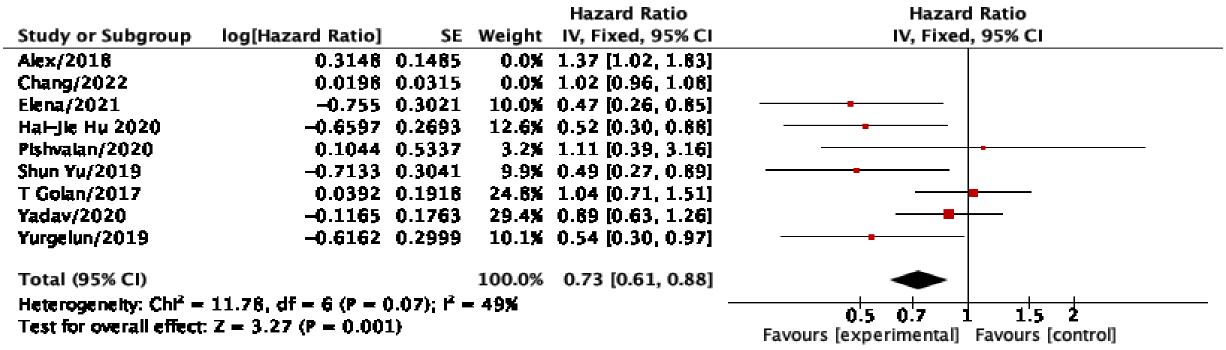

Supplement: Supplementary file 1 [file Image_1.pdf]
